# Serological characteristics of autoimmune pancreatitis and its differential diagnosis from pancreatic cancer by using a combination of carbohydrate antigen 19-9, globulin, eosinophils and hemoglobin

**DOI:** 10.1371/journal.pone.0174735

**Published:** 2017-04-03

**Authors:** Tianlian Yan, Yini Ke, Yi Chen, Chengfu Xu, Chaohui Yu, Youming Li

**Affiliations:** Department of Gastroenterology, The First Affiliated Hospital, Zhejiang University School of Medicine, Hangzhou, China; Medical University Graz, AUSTRIA

## Abstract

Autoimmune pancreatitis (AIP) is a special type of chronic pancreatitis, which may be misdiagnosed as pancreatic carcinoma. This study aims to verify new biomarkers for AIP and propose a serological pattern to differentiate AIP from pancreatic adenocarcinoma with routinely performed tests. In this study, data of serum samples were collected and compared between 25 patients with AIP and 100 patients with pancreatic carcinoma. Receiver operating characteristic analysis and logistic regression was performed to evaluate the diagnostic effect of serum parameters in differentiating AIP from pancreatic carcinoma alone or in combination. Among several serum markers observed in the two groups, carbohydrate antigen 19–9 (Ca19-9), globulin, eosinophils and hemoglobin were selected as the independent markers. Serum levels of Globulin, Eosinophil percentage in AIP group were significantly higher than in pancreatic cancer group (P<0.05), while hemoglobin and tumor marker CA19-9 levels were lower (P <0.05). The combination of these markers identified patients with AIP with 92% sensitivity and 79% specificity, which indicated relatively high diagnostic value. Elevated serum eosinophils, globulin, together with decreased hemoglobin level can be used as a preoperative indicator for AIP and can help to initiate diagnosis of AIP in time.

## Introduction

Since first proposed by Yoshida et al. in 1995 [1], autoimmune pancreatitis (AIP) has gradually become recognized as a unique kind of chronic pancreatitis. Frequently, patients with AIP are characterized clinically by painless obstructive jaundice, histologically by a lymphoplasmacytic infiltration with fibrosis, and therapeutically by a dramatic response to steroids [2, 3]. According to their histopathological differences, AIP is classified into two subtypes [3]. Type 1 AIP is considered to be the pancreatic manifestation of a systemic chronic inflammatory disorder named IgG4-related disease (IRD) [4], whose etiology and pathogenesis remains unclear. In the worldwide, type 1 is more often observed, accounting for 96% of Asian and 80% of European AIP cases [5], whereas type 2 is rare. Consequently, the AIP discussed below is referred to type 1 unless otherwise stated.

Currently, the International Consensus Diagnostic Criteria (ICDC) for AIP is based on pancreatic imaging, serology, extrapancreatic organ involvement, histology and steroid treatment effects [3]. Notably, both AIP and pancreatic malignancy occur mainly in elderly males. AIP and pancreatic cancer patient share many features in common, such as painless jaundice, weight loss, abdominal discomfort, elevated level of serum tumor markers, and diffuse or focal enlargement of parenchymal imaging[6–8]. However, treatment and prognosis of the two separate entities are completely different. Similarity of epidemiology, clinical manifestation and radiological exhibition makes it difficult to differentiate AIP from pancreatic cancer. In our AIP cohort, 56% of out-patients with AIP were misdiagnosed as pancreatic cancer and admitted to the Hepatopancreatobiliary Surgery Department at first. The consequence of misdiagnosing pancreatic cancer for AIP can be devastating, while misdiagnosing AIP for cancer and carrying out an unnecessary pancreatoduodenectomy is also unsatisfactory. Unfortunately, 2.5–11% of patients undergoing pancreatoduodenectomy for presumed pancreatic malignancy are ultimately confirmed as AIP [9–11].

Therefore, exploring serological markers to distinguish AIP from pancreatic cancer is essential. To date, the clinical features of AIP stratified by serum markers have not been fully investigated. Among the serum markers, IgG4 concentration has the highest diagnostic value with sensitivity of 67%-95% and a specificity of 89%-100% [12–15]. However, test of serum IgG4 is rarely performed unless assuming AIP, which depending on the physicians’ clinical experience and their familiarity of the disease. Moreover, 10% of patients with pancreatic carcinoma had elevated serum IgG4 (>140 mg/dL), and in 1–2.4% it was elevated to twice the upper limit of normal, which makes IgG4 insufficient to be the golden diagnostic standard in the setting of obstructive jaundice/pancreatic mass [13, 16, 17]. Considering the limitation of IgG4 test, obtaining new evidence from routinely performed test results can certainly be helpful for primary diagnosis.

In this retrospective large-scale case control study, we aimed to identify serum markers from routinely performed tests and evaluate the diagnostic ability of these serum markers alone and combined in distinguishing AIP from pancreatic adenocarcinoma (PAC). On this basis, we hope to provide evidence and develop a strategy to apply results of routinely performed tests to do the differential diagnosis on the early phase.

## Materials and methods

### Study design and subjects

Study subjects were selected from patients who were hospitalized with the final diagnosis of AIP/PAC in The First Affiliated Hospital, Zhejiang University School of Medicine, China from November 2009 to April 2014.

Diagnosis of AIP was based on the International Consensus Diagnostic Criteria in 2011[3] and all the subjects (n = 25) who met the diagnosis criteria were enrolled. Considering the total number of PAC patients greatly exceeded the number of AIP patients, we randomly selected 100 patients with PAC, whose diagnosis was confirmed by pathological results. Patients diagnosed as AIP or PAC for the first time in the other hospitals were excluded considering a lack of data. For the subjects hospitalized for multiple times in our hospital due to recurrent of AIP or adjuvant chemotherapy/radiation of PAC, only information of the first diagnosis were enrolled.

Data of general conditions, clinical symptoms, serum markers, radiological findings, pathological results and treatment was collected for further statistically analysis. Subjects’ data collection was conducted from March 4^th^ to April 1^st^ 2016.

The protocol was approved by the institutional review board at Zhejiang University and conducted in accordance with the Declaration of Helsinki. Written informed consent was obtained from all subjects. No authors had access to information that could identify individual participants during or after data collection. For data request, please contact the ethics committee of Zhejiang University, School of medicine, the first affiliated hospital, email: yixuelunli123@163.com.

### Laboratory measurements

All of the serum parameters were measured simultaneously as the routinely performed test on the first day of hospitalization. Blood samples were obtained from an antecubital vein and the samples were used for the complete blood cell count, biochemical parameter analysis and electro-chemiluminescense immunoassay (ECLIA). Complete blood cell count was measured using XE2100 (Sysmex, Japan). Parameters included white blood cell count, hemoglobin level (Hb), percentage of eosinophil (E%) and neutrophil percentage were collected. Biochemical parameters were measured by an Olympus AU640 auto-analyzer (Olympus, Kobe, Japan) under standard protocols. These biochemical parameters included total bilirubin (TBIL), direct bilirubin (DBIL), indirect bilirubin (IBIL), alkaline phosphatase (ALP), alanine aminotransferase (ALT), aspartate aminotransferase (AST),γ-glutamyltransferase (GGT). Carbohydrate antigen 19–9 (Ca 19–9) level was measured by ECLIA on a Modular Analytics E module (Roche Diagnostics Co., Tokyo, Japan).

### Statistical analyses

Statistical analyses were performed using SPSS software version 22.0 (IBM, New 9 York, USA). Continuous variables were expressed as a median value with interquartile range (IQR). The Kolmogorov–Smirnov test was used to detect whether continuous data were normally distributed. For comparisons of continuous data the student’s t-test or Mann–Whitney U-test were adopted depending on their normality, while chi-squared test was applied to comparisons of categorical variables. Receiver operating characteristic (ROC) analysis was conducted to assess optimal cut-off levels of Ca 19–9, eosinophil percentage, globulin and hemoglobin. Test characteristics of each parameter and their combination were calculated. Logistic regression analysis was applied to study the simultaneous effect of Ca 19–9, eosinophil percentage, globulin and hemoglobin. False discovery rate (FDR) control was conducted to avoid making a type I error in multiple testing. The p values < 0.05 (two-tailed) were considered statistically significant. FDR adjusted p value< 0.05 were considered that 5% of significant tests will result in false positives.

## Results

### Baseline characteristics

At baseline, 25 patients with AIP and 100 patients with PAC were enrolled. Characteristics of study subjects and serological parameters are shown in **Table 1**.

**Table 1:** Characteristics of subjects.

**Variable**	**AIP (n = 25)**	**PAC (n = 100)**	***p* value**
**Age(year)**	66(58–70)	59 (52–65)	0.033^a^
**Male[n%]**	21 (84%)	69 (69%)	0.212^b^
**Ca19-9 (U/ml)**	23.2(7.6–113.1)	349.8(24.2–1964.6)	<0.001^a^
**E%**	5.4(1.8–6.5)	2.0(1.3–3.0)	0.001^a^
**Hb (g/L)**	Male 114 (105–131)	Male 133(121–144)	0.006^a^
	Female 107 (94–115)	Female 127 (109–132)	0.020^a^
**Globulin (g/L)**	34.4(26.1–39.0)	23.1(25.6–28.3)	<0.001^a^
**TBIL (umol/L)**	33.0 (9.5–117.0)	13.0 (10.0–20.0)	0.190^a^
**DBIL (umol/L)**	16.0 (4.0–72.5)	5.0 (3.0–9.0)	0.017^a^
**ALP (U/L)**	281.0 (112.0–402.5)	85.5 (68.3–150.5)	<0.001^a^
**ALT (U/L)**	109.0 (41.0–486.4)	25.0 (13.0–60)	<0.001^a^
**AST (U/L)**	80.0 (28.5–196.5)	22.5(18.0–41.3)	<0.001^a^
**GGT (U/L)**	272.0 (48.5–569.5)	37.0 (21.0–178.25)	<0.001^a^

Age and laboratory tests in median (interquartile range). *AIP* autoimmune pancreatitis, *PAC* pancreatic adenocarcinoma, *E%* eosinophil percentage, *Hb* hemoglobin, *TBIL* total bilirubin, *DBIL* direct bilirubin, *ALP* alkaline phosphatase, *ALT* alanine aminotransferase, *AST* aspartate aminotransferase, *GGT* γ-glutamyltransferase.

a Mann-Whitney U-test

b Fisher’s exact test

According to the International Consensus Diagnostic Criteria of AIP, all patients in AIP group were diagnosed as type 1 AIP, including 21 males and 4 females. In PAC group, male accounts for 69% of 100 patients. Both occurring predominantly in elderly man, we found no significant gender difference between AIP and PAC group, whereas the onset age of AIP (median 66, IQR 58–70) was significantly higher than that of PAC (median 59, IQR 52–65), p = 0.033.

Except for total bilirubin, all the serological parameters listed in **Table 1**showed significant differences between AIP group and PAC group. Eosinophil percentage, Globulin, DIBL and liver enzymes including ALP, ALT, AST, GGT were significantly higher in AIP group than in PAC group (all p <0.05). In contrast, serum Ca 19–9 and hemoglobin level were significantly lower in AIP than in PAC (all p <0.05).

### Subgroup analysis

In respect that bilirubin and liver enzyme levels are closely related to the clinical manifestations of obstructive jaundice, we further studied distributions of TBIL, DBIL, ALP, ALT, AST and GGT within subgroups of AIP and PAC patients.

Jaundice presented more frequently in AIP group (64%) than PAC group (18%), p<0.001. Consistent with this, levels of bilirubin and liver enzyme were significantly higher in subgroup with jaundice than that without jaundice in both AIP and PAC patients, except for ALT and AST in AIP group, which presented similar trends but not significant in statistics (**Table 2**).

**Table 2:** Bilirubin and liver enzymes le0076els in subgroups of AIP and PAC.

**Variable**	**Jaundice present**	**Jaundice absent**	***p* value**
**AIP**			
**Jaundice [n%]**	16 (64%)	9 (36%)	-
**TBIL (umol/L)**	47.5 (15.8–237.3)	8.0 (7.0–9.0)	0.004
**DBIL (umol/L)**	28.10 (9.8–153.0)	4.0 (3.0–11.0)	0.005
**ALP (U/L)**	304.5 (240.3–414.5)	106.0 (83.5–236.0)	0.017
**ALT (U/L)**	149.5 (57.5–397.25)	54.0 (23.0–275.5)	0.169
**AST (U/L)**	126.5 (48.0–215.8)	71.0(26.5–145.0)	0.452
**GGT (U/L)**	444.5 (229.8–866.75)	59.0 (18.5–406.5)	0.032
**PAC**			
**Jaundice [n%]**	18 (18%)	82 (82%)	-
**TBIL (umol/L)**	251.0 (168.0–316.0)	12.0 (10.0–14.0)	<0.001
**DBIL (umol/L)**	216.0 (153.0–260)	6.0 (4.0–9.8)	<0.001
**IBIL (umol/L)**	89.0 (56.0–131.0)	7.0 (10.0–15.0)	<0.001
**ALP (U/L)**	316.0 (224.0–523.0)	79.0(64.0–98.5)	<0.001
**ALT (U/L)**	126.0 (56.0–163.0)	19.0 (11.0–35.5)	<0.001
**AST (U/L)**	80.0 (33.0–130.0)	20.0(17.0–27.5)	<0.001
**GGT (U/L)**	450.0 (210.0–789.0)	25.0 (19.0–60.5)	<0.001

Laboratory tests in median (interquartile range). Mann-Whitney U-test. *AIP* autoimmune pancreatitis, *PAC* pancreatic adenocarcinoma, *TBIL* total bilirubin, *DBIL* direct bilirubin, *IBIL* indirect bilirubin, *ALP* alkaline phosphatase, *ALT* alanine aminotransferase, *AST* aspartate aminotransferase, *GGT* γ-glutamyltransferase.

**Fig 1:**
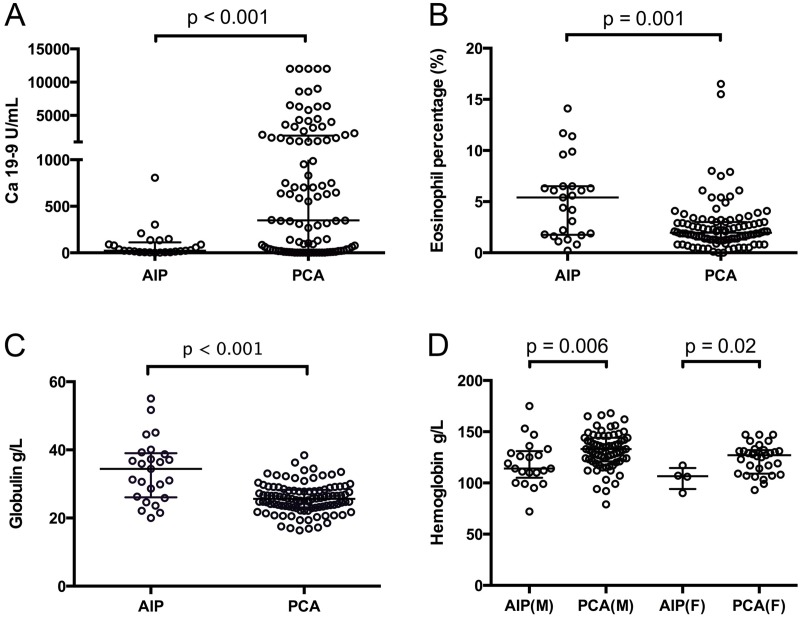
Comparison of serological parameters in patients with autoimmune pancreatitis (AIP) and pancreatic adenocarcinoma (PAC). In all the panels, each circle represents a measurement for one patient. Levels of each parameter are expressed as median (horizontal bar in the middle) and interquartile range (within 75th percentile and 25th percentile bars from upper to lower). P values are obtained from Mann–Whitney U test. *AIP* autoimmune pancreatitis, *PAC* pancreatic adenocarcinoma. *AIP(M)* autoimmune pancreatitis in male patients, *AIP(F)* autoimmune pancreatitis in female patients, *PAC(M)* pancreatic adenocarcinoma in male patients, *PAC(F)* pancreatic adenocarcinoma in female patients.

Considering the heterogeneity within the AIP and PAC groups, bilirubin and liver enzymes were excluded. Thus, other markers including serum Ca 19–9, eosinophil, globulin and hemoglobin were selected as serum markers to identify AIP or PAC. Distributions of the selected serum markers were shown in **Fig 1A–1D**, respectively.

### Serological characteristics of AIP and PAC

High level of Ca 19–9 was independently associated with PAC (72%), compared with AIP (44%) [Odds ratio (OR) 0.306; 95% Confidence interval (CI) 0.124–0.753, p = 0.016]. In AIP group, elevation of eosinophil percentage (52%) was more frequently observed than PAC group (10%) [OR 9.751, 95%CI 3.512–27.066, p < 0.001]. Decrease of hemoglobin level appeared more frequently in AIP group (76%) than PAC group (40%) [OR 4.750, 95%CI 1.746–12.928, p = 0.002]. Also, significant differences in frequency of serum globulin elevation was detected in AIP group (48%) and PAC group (2%) [OR 45.231, 95%CI 9.088–225.119, p <0.001] **(Table 3**). All the elevation or reduction of serum level were determined by normal reference values.

**Table 3:** Elevation or decease frequency of serum markers.

**Variable**	**AIP (n = 25)**	**PAC (n = 100)**	**OR (95%CI)**	***P* value**
**Elevated Ca19-9**	11 (44%)	72 (72%)	0.306 (0.124–0.753)	0.016
**Elevated E%**	13 (52%)	10 (10%)	9.751 (3.512–27.066)	<0.001
**Decreased Hb**	19(76%)	40 (40%)	4.750 (1.746–12.928)	0.002
**Elevated Globulin**	12 (48%)	2 (2%)	45.231 (9.088–225.119)	<0.001

Chi-square test. Elevation or decrease of serological parameters were determined by normal reference value: Ca19-9 0–37 U/mL; Eosinophil percentage 0.5%-5%; Hemoglobin: Male 131–172 g/L, Female 113–151 g/L; Globulin: 20–35 g/L. *OR* odds ratio, *CI* confidence interval, *E%* Eosinophil percentage

### Diagnostic characteristics of serological parameters used singly

**Fig 2:**
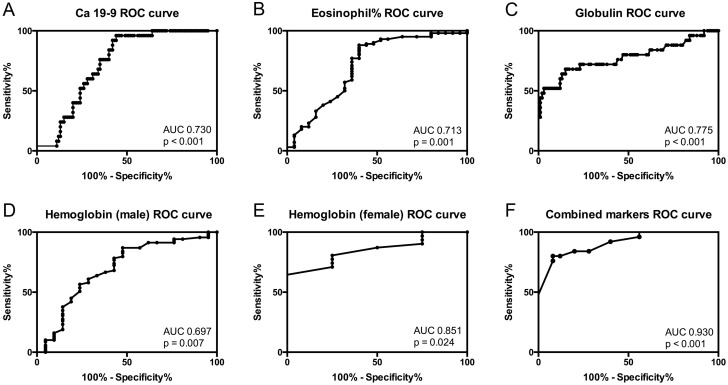
**Receiver operating curve** of A. Ca 19–9, B. Eosinophil percentage, C. Globulin level, D. Hemoglobin (male), E. Hemoglobin (female) and F. Combined four serological markers.

We performed receiver operating characteristic (ROC) curves for differentiating AIP from PAC based on Ca 19–9, eosinophil percentage, globulin and hemoglobin (**Fig 2A–2E**). As shown in **Table 4**, the area under the curve (AUC) for Ca 19–9 to diagnose AIP from PAC was 0.730 at an optimal cut-off level of 306.75 U/ml (Ca19-9<306.75u/ml indicated AIP, if not PAC was assumed), yielding a sensitivity of 56% and a specificity of 96%. Eosinophil percentage at the cut-off 4.15% (E%>4.15% indicated AIP) showed the sensitivity of 60% and specificity of 88%, with AUC reaching to 0.713. The sensitivity and specificity of globulin to distinguish AIP from PAC were 68% and 85% respectively at the optimal cut-off level 29.8 g/L (Globulin>29.80 g/L indicated AIP), with AUC to be 0.775. The optimal cut-off for hemoglobin level in male and female were calculated separately, due to its significant gender differences. The optimal cut-off for hemoglobin in male and female patients were determined as 114.5 g/L (hemoglobin <114.5 g/L indicated AIP, sensitivity 87%, specificity 52%) and 118.5g/L (hemoglobin <118.5 g/L, sensitivity 65%, specificity 100%), encompassing the AUC of 0.697 and 0.851, respectively. False positive rate (FDR) control was conducted to adjust p value when conducting logistic regression and ROC analysis. FDR < 0.05 was achieved with regard of all the parameters listed in **Table 4.**

**Table 4:** Diagnostic performance of serological parameters singly and combined, to differentiate AIP and PAC.

**Variable**	**AUC(95%CI)**	***P* value**	**FDR**	**Sensitivity**	**Specificity**
**Ca19-9 < 306.75 u/ml**	0.730 (0.643–0.817)	<0.001	<0.0015	56%	96%
**E% > 4.15**	0.713 (0.584–0.843)	0.001	0.0017	60%	88%
**Globulin > 29.80 g/L**	0.775 (0.651–0.899)	<0.001	<0.0015	68%	85%
**Hb (M) < 114.5 g/L**	0.697 (0.557–0.836)	0.007	0.0084	87%	52%
**Hb (F) < 118.5 g/L**	0.851 (0.701–1.000)	0.024	0.0240	65%	100%
**Combination**	0.930 (0.880–0.979)	<0.001	<0.0015	92%	79%

*E%* eosinophil percentage, *Hb* hemoglobin, *FDR* False positive rate

### Diagnostic model of combined serum parameters

To explore a satisfactory diagnostic model to distinguish diseases, Logistic regression analysis was performed by using serum Ca 19–9, eosinophil percentage, globulin and hemoglobin, which were significantly increased or decreased in AIP patients compared with PAC patients. In the Logistic regression analysis, these four variables were transformed to score 1 or 0 using the optimal cut-off and combined to form a new predicted value. Predicted value >0.1066105 suggested AIP while predicted value <0.1066105 indicated PAC, when applying the specific score into the function (**Table 5**): Predicted value = 1/[1+e^-(-6.091+3.247X1+2.412X2+1.940X3+1.768X4)^].

**Table 5:** Results of the logistic regression analysis for AIP.

Variable	β	SE	Wald value	*P* value	OR	95% CI of OR
X1 (Ca 19–9)	3.247	1.121	8.392	0.004	25.720	2.858–231.430
X2 (E%)	2.412	0.737	10.712	0.001	11.155	2.632–47.287
X3 (Globulin)	1.940	0.684	8.038	0.005	6.956	1.820–26.588
X4 (Hemoglobin)	1.768	0.724	5.963	0.015	5.859	1.417–24.216
Constant	-6.091	1.280	22.660	<0.001	0.002	-

β: partial regression coefficient; SE: standard error of partial regression coefficient; OR: odds ratio; CI: confidence interval

P = 1/[1+e^-(-6.091+3.24X1+2.412X2+1.940X3+0.724X4)^]

X1 = 1: Ca19-9<306.75u/ml; X1 = 0: Ca19-9>306.75u/ml

X2 = 1: E%>4.15%; X2 = 0: E%<4.15%

X3 = 1: Globulin>29.80 g/L; X3 = 0: Globulin<29.80 g/L

X4 = 1: Hb (M) < 114.5 g/L; X4 = 0: Hb (M) > 114.5 g/L

Hb (F) < 118.5 g/L; Hb (F) > 118.5 g/L

### Combination of four serum parameters increases diagnostic value for AIP

To confirm the diagnostic accuracy of the functions, our cohort with 25 AIP patients and 100 PAC patients were predicted using the functions.

Logistic regression analysis showed relatively high sensitivity and specificity (92% and 79%) in AIP diagnosis, with AUC reaching to 0.930 (P<0.001), as shown in **Fig 2F**.

## Discussion

In this study, we reported concentration differences of the four serum markers, Ca 19–9, eosinophil percentage, globulin and hemoglobin, in patients with AIP and pancreatic adenocarcinoma. We hope that this research will provide additional evidence to differential diagnosis between the two diseases. Serum levels of globulin, eosinophil percentage in AIP group were significantly higher than those in pancreatic cancer group (p<0.05), while hemoglobin and tumor marker CA19-9 levels were significantly lower (p<0.05). In a single test, none of these serological markers was sufficient for diagnosis. However, the combination of these four could identify AIP with sensitivity of 92% and specificity of 79%, which indicates a relatively high diagnostic value.

**Fig 3:**
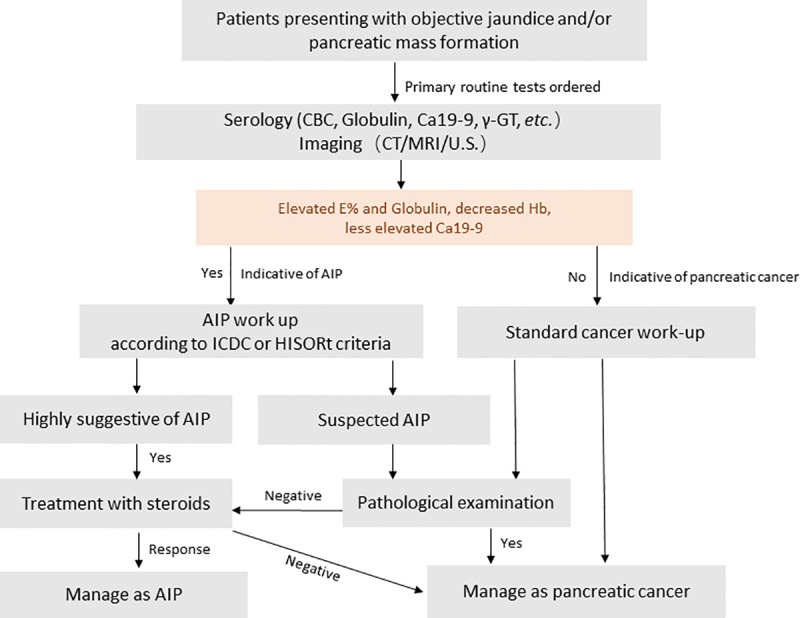
An algorithm of clinical pathway for distinguishing autoimmune pancreatitis from pancreatic cancer. Strategy for initiation of AIP wok-up is based on combined analysis of Ca19-9 level, Eosinophil percentage, Globulin and Hemoglobin level. Elevated eosinophil percentage, globulin level together with decreased hemoglobin level are suggestive of AIP. According to our present study, Ca19-9 < 306.75 u/ml, E%>4.15%, Globulin>29.80 g/L and Hb (M) < 114.5 g/L Or Hb (F) < 118.5 g/L are suggested to be the cut-off value for diagnosis. Abbreviations: AIP, autoimmune pancreatitis; ICDC, International Consensus Diagnostic Criteria for AIP; HISORt, histology, imaging, serology, other organ involvement, response to steroid therapy criteria for autoimmune pancreatitis.

Although our study is not the first one to address diagnostic ability of a combination of serological markers, we found that this is the first study to apply serum eosinophil and hemoglobin levels in differential diagnostic tests. As routinely performed tests, results of complete blood count and clinical biochemistry can provide early phase of evidence and initiate diagnosis of AIP in the settings of jaundice or mass formation. When patients with suspected pancreatic malignancy come to visit, a review of the imaging data is essential if globulin, eosinophil levels rise and hemoglobin decreases. A subsequent serum IgG4 test should be ordered if the typical imaging features of AIP are disclosed [18]. Here we put forward a clinical pathway regarding patients presenting with objective jaundice or mass formation. Strategy for initiation of AIP wok-up is based on combined analysis of Ca19-9 level, Eosinophil percentage, Globulin and Hemoglobin level[3, 19] (**Fig 3**).

From a serological point of view, pathogenesis of AIP can be attributed to autoimmunity, as indicated by specific serologic abnormalities and the dramatic response to steroid therapy[20]. AIP patients show high incidences of hypergammaglobulinemia (43%), increased levels of IgG (62–80%), IgG4 (68–92%) and antinuclear antibodies (40–64%), rheumatoid factor (25%) [14, 21, 22]. Elevated levels of serum amylase and lipase[23], B cell-activating factor[24], autoimmune markers such as anti-carbonic anhydrase II antibodies, antimitochondrial antibodies have also been reported in patients with AIP[25]. Among them serum IgG4 presents highest sensitivity of 67%-95% and specificity of 89%-100% at varied cut-off value determined by reporters[12–15]. In ICDC, serum IgG4 level more than 2 times the normal upper limit is strongly suggestive for diagnosis of AIP[3].

Apart from autoimmune antibodies, other novel markers including serum micro RNAs (miRNAs) signature have been reported to play a role in differentiating pancreatic carcinoma from AIP with high accuracy [26, 27]. MiRNA are endogenous small non-coding RNAs consisting of 19–25 nucleotides, which can interfere with gene transcription and/or translation, thus modulate the targeted gene expression[28]. Till now, large numbers of miRNAs are implicated in the pathogenesis and progression of pancreatic cancer, including *KRAS*-related miRNAs (*e*.*g*. miR-217), TGF-β-related miRNAs (*e*.*g*. miR-483), miR-155, miR-21, miR-34 and so on[28]. They not only provide for novel therapeutic options for pancreatic malignancy treatment, also the chance to aid in early diagnosis, disease monitoring and prognostic analysis[27, 29]. Recently, Manabu Akamatsu and his colleagues reported that four MAKP-associated miRNAs, miR-7, miR-34a, miR-181d, and miR-193b can be candidate biomarkers to differentiate pancreatic malignancy from AIP [30]. In this work, significantly higher amounts of serum miRNAs were detected in patients with pancreatic ductal adenocarcinoma (PDCA) than in those with AIP, and sensitivity of 72–79% together with specificity of 73–80% were obtained in ROC curve analysis [30]. In recent work of Johansen and his colleagues, they analysed serum level of 34 miRNAs in patients with pancreatic cancer, chronic pancreatitis and healthy controls and developed a diagnostic panels based on 5 or 12 miRNAs (miR-16, -18a, -20a, -24, -25, -27a, -29c, -30a.5p, -191, -323.3p, -345 and miR-483.5p) to distinguish pancreatic cancer from pancreatitis. When combined with serum CA 19–9, the highest AUC reached 0.94 (0.90–0.97) [27]. In conclusion, MicroRNAs regulate expression of more than 60% of all mammalian protein -coding genes. Serum miRNAs have been suggested as promising biomarkers to distinguish patients with pancreatic cancer from non-malignant diseases. Further investigations are needed to clarify expression profiles of miRNAs in AIP and differential diagnostic capacity of circulating miRNAs in AIP/PAC.

Eosinophil infiltration has been observed in IgG4-related disease, pancreatic carcinoma and chronic pancreatitis[31, 32], but eosinophils >10/HPF was more frequently seen in AIP[33]. However, the severity of histological infiltration did not correlate with peripheral eosinophilia[34]. We reported that 52% patients showed elevated serum eosinophil percentage, which is relatively high compared to the previous reports of 12–45% measured by periphery eosinophil counts[32, 35, 36]. Eosinophil percentage at an optimal cut-off level 4.15% diagnosed AIP with the sensitivity of 60% and specificity of 88%, which indicated a relatively high diagnostic performance. The occurrence of eosinophilia during course of AIP may not reflect an allergic phenomenon, but may be related to autoimmune mechanisms, serous membrane response, or the progression of pancreatic inflammation and fibrosis [32].

Mild malnutrition as well as impaired pancreatic endocrine/exocrine function are commonly observed in patients with AIP and pancreatic carcinoma. In our study, we also found that mild anemia occurred in both AIP and PAC group, with more frequent decrease and lower level of hemoglobin observed in patients with AIP. Taking the histopathological features of type1 AIP into consideration, it is reasonable to speculate that autoimmunity plays a role in anemia in patients with AIP. In recent years, autoimmune hemolytic anemia (AIHA) has been reported to coexist with AIP, IgG4-related sclerosing cholangitis (IgG4-SC), primary sclerosing cholangitis (PSC) and other autoimmune disorders[37–40]. We hypothesize that in patients with type 1 AIP, chronic erythrocyte-lysis and hemolytic process take place in the same way in which AIHA is developed. Thus, increased IgG antibodies, especially IgG1 and IgG3, bound to erythrocyte membranes might be identified by IgG Fc receptors of phagocytes and phagocytized. Then the complement activation process ultimately leads to erythrocyte lysis and mild anemia[39]. In respect that IgG4 is known to be incapable of complement activation [41], it may be other IgG members but not IgG4 which contribute to the development of IgG4-related anemia or AIHA. As for the patients with IgG4- related tubulointerstitial nephritis (TIN), mild normochromic normocytic anemia may result from kidney function abnormalities[42, 43].

The limitation of this study may come in four ways. Firstly, diagnosis of AIP was based on ICDC[3], which means that AIP patients were not always confirmed by histopathological examination. Patients with AIP ultimately progressing to pancreatic carcinoma or cases where AIP coexisted with pancreatic carcinoma were reported[44]. Thus, we cannot absolutely rule out the occult pre-malignancy and malignancy in AIP patients without histological and extensive follow up. Secondly, in our single-center retrospective study, the serum IgG4 levels of patients enrolled before 2011 were not available. Thus, further validations for our study by adopting or comparing with serum IgG4 levels are needed. Thirdly, we recruited only the type 1 AIP into AIP group and pancreatic adenocarcinoma into pancreatic carcinoma group. Actually, for type 2 AIP, serological investigation is more urgently needed because of limited values of autoimmune markers and IgG4. Further study is needed to investigate the diagnostic parameters to discriminate type 2 AIP from pancreatobiliary malignancy. Also, as a retrospective study we enrolled only 25 patients in the AIP group, which is relatively a small sample. Lack of healthy controls and other pancreatic disease controls such as chronic pancreatitis in our study might cause overestimation of the quality of the tests in disease with low prevalence[45]. Large-scale, multi-centered prospective trials are needed to confirm the results of this study in the future.

## Conclusion

In this study, we reported serological characteristics of AIP and described a strategy of differential diagnosis at an early phase by using a combination of four serological markers. Elevated serum eosinophil and globulin levels together with decreased hemoglobin level can be used as a preoperative indicator for AIP and can help to avoid unnecessary operation. Multi-center collaboration is needed so as to recruit an adequate sample of subjects and obtain stable and convincing results. Also, the mechanisms of extensive anemia presented in patients with AIP need further investigation, which may implicate the pathogenesis of AIP in a serological aspect.

## Supporting information

S1 File Abbreviations. Abbreviations for the repeated terms.
(DOCX)

S2 File STROBE_checklist_PONE_S_16_46884. STROBE checklist for the present cohort study.
(DOCX)
