# Supplementary material for: Serological characteristics of autoimmune pancreatitis and its differential diagnosis from pancreatic cancer by using a combination of carbohydrate antigen 19-9, globulin, eosinophils and hemoglobin
Source: PLoS One. 2017 Apr 3;12(4):e0174735. doi: 10.1371/journal.pone.0174735 (PMC5378371; doi:10.1371/journal.pone.0174735)
Supplement: S1 File — Abbreviations for the repeated terms. (DOCX) [file pone.0174735.s001.docx]

Abbreviation:

alanine aminotransferase ALT

alkaline phosphatase ALP

aspartate aminotransferase AST

autoimmune hemolytic anemia AIHA

autoimmune pancreatitis AIP

carbohydrate antigen 19-9 Ca19-9

direct bilirubin DBIL

γ-glutamyltransferase GGT

hemoglobin level Hb

IgG4-related disease IRD

indirect bilirubin IBIL

International Consensus Diagnostic Criteria ICDC

pancreatic adenocarcinoma PAC

percentage of eosinophil E%

total bilirubin TBIL

receiver operating characteristic ROC
